# Expression level of the growth factor progranulin is related with development of systemic lupus erythematosus

**DOI:** 10.1186/1746-1596-8-88

**Published:** 2013-05-23

**Authors:** Feng Qiu, Lijun Song, Feng Ding, Huaxiang Liu, Qiang Shu, Ning Yang, Weiwei Liu, Xingfu Li

**Affiliations:** 1 Department of Rheumatology, Qilu Hospital, Shandong University, Jinan 250012, P. R. China; 2 Department of Rheumatology, Qilu Hospital, Shandong University, No. 107, Wenhua Xi Road, Jinan 250012, P. R. China

**Keywords:** Systemic lupus erythematosus, Progranulin, Glucocorticoid, IL-6

## Abstract

**Background:**

This study is to investigate the expression of progranulin (PGRN) in systemic lupus erythematosus (SLE) patients and the effect of glucocorticoid (GC) treatment on its expression.

**Methods:**

Thirty newly diagnosed severe SLE patients and 30 healthy subjects were enrolled in this study. The serum levels of PGRN and the inflammatory factors of SLE were detected by ELISA and the mRNA expression of these proteins were detected by real-time PCR.

**Results:**

The serum levels of PGRN, IL-6, PR3, TNFR, TNF-α and anti-dsDNA antibody in SLE patients were increased significantly compared with healthy controls (P < 0.05). The relative expression of PGRN mRNA was increased by 4.88-fold in pre-treatment SLE patients compared with controls (P < 0.05). After prednisone treatment, the serum levels of PGRN decreased significantly, and the relative expression of PGRN mRNA was decreased by 1.34-fold compared with the untreated controls (P < 0.01). Moreover, Serum concentration of PGRN was correlated with serum levels of IL-6, TNF-α, TNFR and anti-dsDNA antibody in both pre-treatment and post-treatment SLE patients.

**Conclusions:**

PGRN is up-regulated in the SLE patients and is correlated with pro-inflammatory cytokines and anti-dsDNA antibody. Glucocorticoids can down-regulate the expression of PGRN in SLE patients.

**Virtual slides:**

http://www.diagnosticpathology.diagnomx.eu/vs/1562484036905973

## Introduction

Systemic lupus erythematosus (SLE) is a prototypic autoimmune disease of unknown origin affecting major organs, which mostly occurred in women of childbearing age. SLE is primarily caused by high levels of autoantibodies and immune complex deposition [1]. In SLE patients, disorder cytokine production induces immunodeficiency and leads to tissue inflammation and organ damage. The progranulin protein (PGRN) is an autocrine growth factor with multiple physiological and pathological functions. Tang W et al. had found that PGRN can bind to TNF receptors and is therapeutic against inflammatory arthritis in mice [2]. Therefore, PGRN is a potential target for the treatment of autoimmune diseases. However, the expression changes of PGRN in SLE patients remains unclear. Glucocorticoid (GC) is an important drug for treatment of SLE. GC inhibits the expression and function of many cytokines though two pathways: the genomic pathway and the non-genomic pathway [3,4]. However, whether GCs could exert their function through affecting the expression of PGRN is need to be studied.

In this study, we tested serum levels and mRNA levels of PGRN, IL-6, proteinase3 (PR3), TNFR, TNF-α in the peripheral blood mononuclear cells (PBMCs) of SLE patient and normal controls and dsDNA antibody to investigate the possible role of PGRN in SLE patients. The possible effects of GCs on PGRN in SLE patients were also determined.

## Materials and methods

### Subjects

Thirty newly diagnosed SLE patients with SLEDAI ≥ 10 were recruited in the present study. All of the patients met the American College of Rheumatology revised criteria in 1997 for the classification of SLE [5]. None of them had been treated with GCs and other immunosuppressive drugs prior to first collection of specimen. All of them received prednisone 1 mg/kg/day for 21 consecutive days. Peripheral blood samples were obtained again 3 weeks after prednisone administration. The control group included 30 sex- and age-matched healthy volunteers (23 females and 7 males, age range 18–59 years, median 30.1 years). All subjects signed informed consent forms. Ethical approval for the research was obtained from the Medical Ethical Committee of Qilu Hospital, Shandong University.

### Quantitative real-time polymerase chain reaction (RT -PCR)

PBMCs were separated by Red Blood Cell Lysis Buffer (Pharmacia Diagnostics, Uppsala, Sweden), and the total RNA was isolated by Trizol Reagent (Invitrogen, America) according to the manufacturer’s instructions. RNA concentration was determined using the Eppendorf Biophotometer (Brinkmann Instruments, Westbury, NY, USA) and normalized to 1 ug/ml for reverse transcription. The cDNA was reverse-transcribed using the ReverTra Ace qPCR RT Kit (Toyobo, Osaka, Japan). Real-time quantitative PCR was performed by Light Cycler TaqMan Master kit (Toyobo, Osaka, Japan) according to manufacturer’s instruction on a Bio-rad IQ5 detection systems (Bio-rad, CA, USA). The primers (Huada, Shanghai, China) used for RT-PCR were shown in Table 1.

**Table 1:** Primers used in this study

**Gene**	**Forward primers (5′-3′)**	**Reverse primers (5′-3′)**
PGRN	gatcctgcgagaaggaagtg	ggccagtaatgcaggct
IL-6	aggagacttgcctggtgaaa	gtactgggaatcggtacg
PR3	ccatgcggcatagctataatt	gacctttattggcgtacttc
TNFR	accaagtgccacaaaggaac	gcggtaccatattaaccgg
GAPDH	cagaacatcatccctgcctctac	ggcattccggtcgtgggc

The following florescent real-time quantitative RT-PCR by using SYBR Green (Toyobo, Osaka, Japan) conditions were used: 95°C for 10 s, followed by 30 cycles of 95°C for 5 s and 60°C for 41 s. Each experiment were performed in triplicate. The PCR products were separated in an agarose gel to confirm the expected size. A melting-curve analysis was also performed to ensure specificity of the products. Relative expression of cytokine mRNAs was determined by comparative Ct method (using arithmetic formulae) by the relative expression software tool (Bio-rad, CA, USA), and the relative expression of the PGRN was calculated using the ΔΔCT method. Expression of mRNAs was normalized to the expression of GAPDH gene.

### ELISA

Five millilitres of heparinized venous peripheral blood and five millilitres coagulation blood were collected from each patient and control subjects before and after the administration of prednisone. The blood was centrifuged and the serum specimens were stored at -80°C. Serum levels of PGRN, IL-6, PR3 and TNFR were measured using a commercial ELISA assay kit (Yonghui, Beijing, China) according to the manufacturer’s instruction.

### Statistical analysis

Statistical analysis was performed using SPSS17.0. Data were presented as median ± IQR. All the data were analyzed with the non-parameter test. The comparisons among pre-treatment, post-treatment and control group were performed by independent sample nonparametric test. The correlations between PGRN and other cytokines or anti-dsDNA antibody were assessed by Spearman rank correlation. P < 0.05 was considered as statistically significant.

## Results

### Clinical characteristics

The clinic-pathological characteristics of the 30 patients enrolled in this study were first analyzed. Among the patients, 23 were females and 7 were males, with ages ranged from 12 to 52 years old (28.4 ± 9.3). The course of disease from the presence of symptoms to the enrollment varied from 1 to 37 months (12.6 ± 10.1). The systemic lupus erythematosus disease activity index (SLEDAI) scores ranged from 10 to 23 (15.1 ± 3.8) of pre-treatment and 6 to 22 (12.2 ± 4.0) of the post-treatment.

### PGRN level was increased in the serum of SLE patients

To investigate the inflammatory effect of SLE, serum levels of inflammatory cytokines, cytokine receptors as well as dsDNAa antibody were detected by ELISA. The levels of PGRN, IL-6, TNFR, TNF-α, PR3 and dsDNA antibody in SLE patients (both pre-treatment and post-treatment) with SLEDAI ≥ 10 were up-regulated significantly compared with that of the normal controls (p < 0.05, Table 2). Therefore, the level of PGRN was changed in SLE patients compared with normal controls.

**Table 2:** Comparison of serum levels of PGRN and other cytokines

**Group**	**n**	**PGRN (pg/l)**	**IL-6 (pg/ml)**	**PR3 (ng/ml)**	**TNFR (ng/l)**	**TNFα (ng/l)**	**Anti-dsDNA antibodies (mg/l)**
Pre-treatment	30	17.82 ± 5.18^#&^	1.56 ± 1.18^#&^	49.33 ± 18.85^#^	17.50 ± 10.33^#&^	89.59 ± 20.27^#&^	208.19 ± 194.24^#&^
Post-treatment	30	13.64 ± 4.51*	0.97 ± 0.37*	48.25 ± 10.89*	10.61 ± 3.70*	54.31 ± 17.17*	137.02 ± 80.71*
Control	30	11.09 ± 1.62	0.70 ± 0.05	38.32 ± 3.55	9.76 ± 1.06	37.46 ± 11.63	23.24 ± 20.23

Note: #, p < 0.05, compared with the normal controls.

&, p < 0.05, compared with the post-treatment group.

*, p < 0.05, compared with the normal controls.

### PGRN mRNA levels were increased in SLE patients

**Figure 1:**
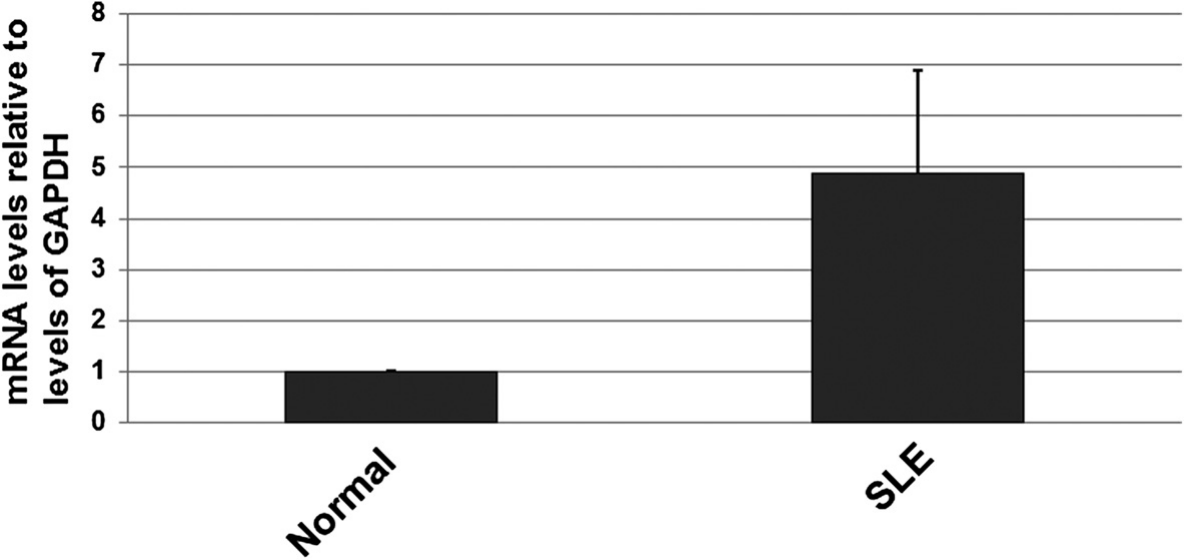
**Quantitative RT-PCR analysis of PGRN mRNA levels. **PBMCs were separated by Red Blood Cell Lysis Buffer (Pharmacia Diagnostics, Uppsala, Sweden), and total RNA was isolated by Trizol Reagent (Invitrogen, America) according to the manufacturer’s instructions. Each sample was run in triplicate. Expression of mRNAs was normalized to the expression of GAPDH gene.

Next, changes in the PGRN mRNA level were determined by real-time PCR. Using the IQ5 software, the data are presented as the fold change in gene expression normalized to GAPDH. The relative expression of PGRN mRNA was increased by 4.88-fold in pre-treatment SLE patients compared to normal controls (P < 0.01) (Figure 1).

### Prednisone treatment downregulated the level of PGRN and inflammatory factors in the SLE patients

Prednisone was reported to decrease the level of IL-6 and TNF-α previously, so we investigated the effect of prednisone on PGRN expression in SLE patients. After treatment, there were significant downregulation in the serum levels of PGRN (P = 0.02), IL-6 (P = 0.022), TNFR (P = 0.003), TNF-α (P = 0.001) and anti-dsDNA antibody (P = 0.038). However, the serum level of PR3 didn’t decreased after treatment (P = 0.549, Table 2).

Relative expression of PGRN mRNA was decreased by 1.34-fold in post-treatment SLE patients compared with pre-treatment ones (P < 0.05). Statistically significant downregulation of IL-6 (P < 0.05), PR3 (P < 0.05), and TNFR (< 0.05) mRNA expressions was also detected in post-treatment SLE patients compared with pre-treatment SLE patients.

### Correlations of PGRN with inflammatory factors in SLE patients

**Figure 2:**
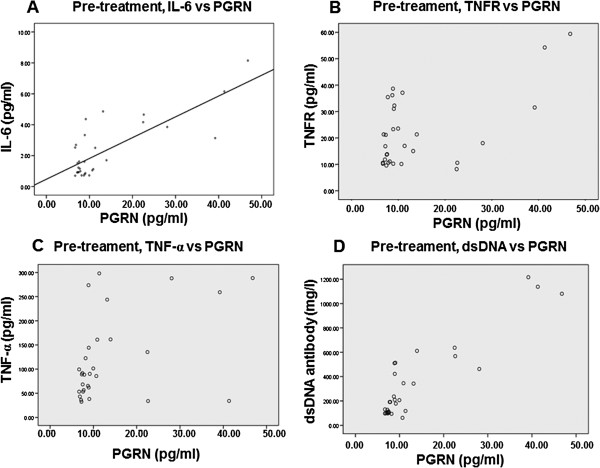
**Spearman rank correlation analysis of PGRN and inflammatory factor in SLE patients before prednisone treatment.** PGRN was correlated with the levels of IL-6 (r = 0.790, P < 0.01), TNFR (r = 0.559, P = 0.01), TNF-α (r = 0.438, P = 0.015) and anti-dsDNA antibody (r = 0.906, P < 0.01) in the serum of pre-treatment SLE patients. (**A**) Pre-treatment, IL-6 vs PGRN; (**B**) Pre-treament, TNFR vs PGRN; (**C**) Pre-treament, TNF-α vs PGRN; (**D**) Pre-treament, dsDNA vs PGRN.

**Figure 3:**
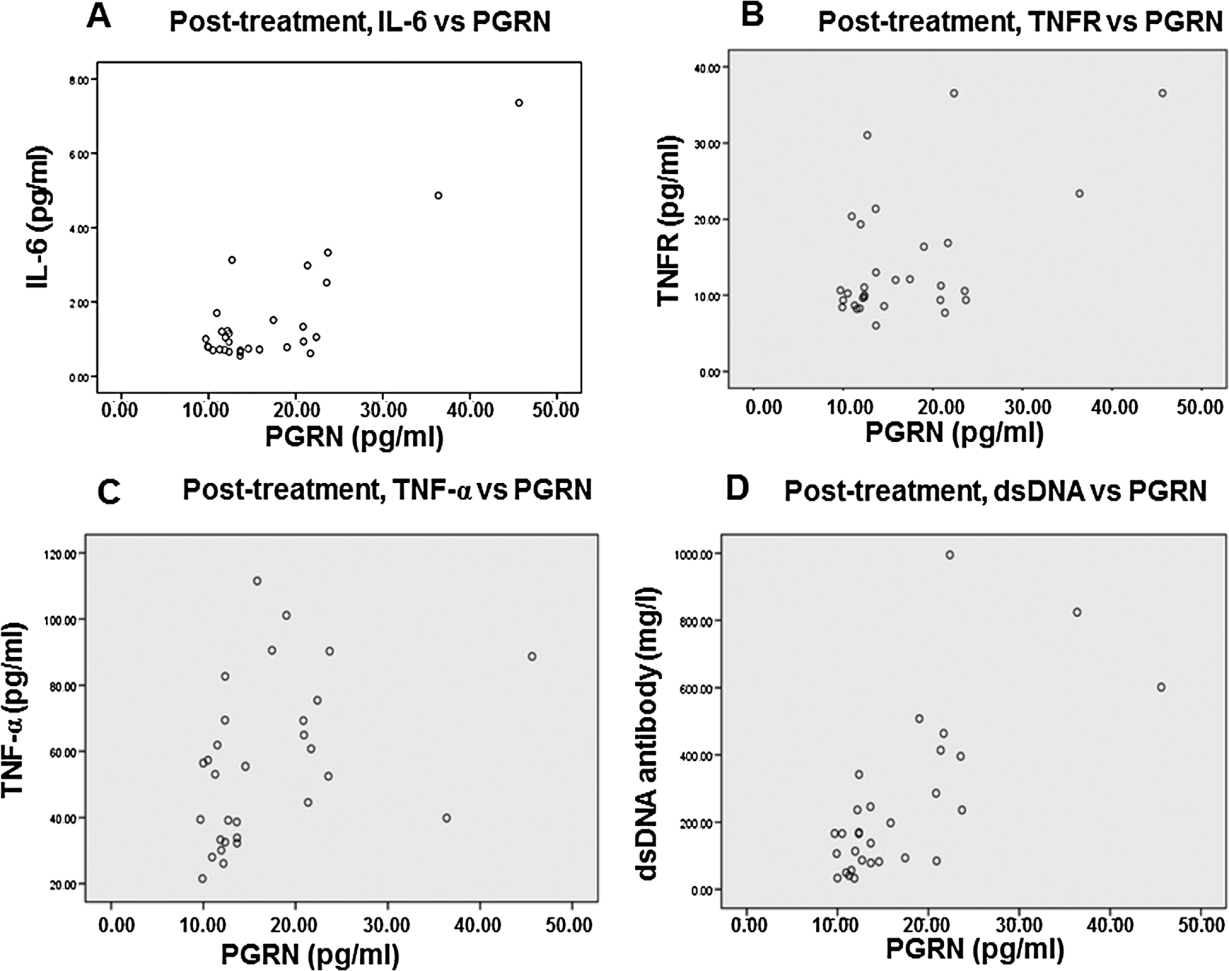
**Spearman rank correlation analysis of PGRN and inflammatory factor in SLE patients after prednisone treatment.** PGRN was correlated with IL-6 (r = 0.836, P < 0.01), PR3 (r = 0.396, P = 0.031), TNFR (r = 0.533, p = 0.02), TNF-α (r = 0.378, P = 0.039) and anti-dsDNA antibody (r = 0.712, P < 0.01) in post-treatment SLE patients. (**A**) Post-treatment, IL-6 vs PGRN; (**B**) Post-treament, TNFR vs PGRN; (**C**) Post-treament, TNF-α vs PGRN; (**D**) Post-treament, dsDNA vs PGRN.

To examine the relationship between serum PGRN level and serum level of SLE-related inflammatory factors, correlation between PGRN and other factors was analyzed by Spearman rank correlation in SLE patients before and after prednisone treatment. Results showed that the serum concentration of PGRN was correlated with the levels of IL-6 (r = 0.790, P < 0.01), TNFR (r = 0.559, P = 0.01), TNF-α (r = 0.438, P = 0.015) and anti-dsDNA antibody (r = 0.906, P < 0.01) in the serum of pre-treatment SLE patients (Figure 2). After treatment of prednisone for 3 weeks, serum concentrations of PGRN in the patients were correlated with IL-6 (r = 0.836, P < 0.01), PR3 (r = 0.396, P = 0.031), TNFR (r = 0.533, p = 0.02), TNF-α (r = 0.378, P = 0.039) and anti-dsDNA antibody (r = 0.712, P < 0.01) in post-treatment SLE patients (Figure 3). No correlation was found between PGRN and PR3 in pre-treatment SLE patients (r = 0.298, P = 0.110, data not shown).

## Discussion

SLE is an autoimmune disease characterized by multiple autoantibodies against self-directed antigens and multisystemic involvement. PGRN is an autocrine growth factor contains seven and a half repeats of a cysteine-rich motif in the order P–G–F–B–A–C–D–E, in which G–E are full repeats and P is the half-motif [6]. PGRN is mainly expressed in epithelial cells, immune cells, neurons [7], and chondrocytes [8]. Moreover, high levels of PGRN expression are found in a variety of human cancers [7]. Several studies have revealed that PGRN plays an important role in many pathological processes, including early embryonic development, wound healing and inflammation [8-13]. PGRN also functions as a regulator of cartilage development and degradation [13], and PGRN exhibites higher affinity for TNF receptors (TNFR), especially TNFR2 when compared with TNF-α [2]. In this study, the level of PGRN and TNFR was also increased in SLE patients. PGRN acts as a powerful antagonist of TNF-α signaling and disturbs the binding of TNF-α and TNFR [2]. Previous researches showed that PGRN convectively suppressed TNF-α-mediated activation of neutrophil granulocyte [12] and degradation of chondrocyte [8]. In summary, PGRN, which binds directly to TNFR, is involved in many physiological and pathological functions. It also plays a critical role in the pathogenesis of inflammatory arthritis in mice. Up-regulation of PGRN has been reported in hemotherapy-induced amenorrhea (CIA) [12]. Our present study showed that the level of PGRN in peripheral blood was upregulated in both pre-and post-treatment SLE patients compared to healthy controls, which is in accordance with the above reports. TNF shows different physiologic and pathogenic effects in autoimmune diseases [14]. TNF has both immuno-suppressive and pro-inflammatory effects in SLE patient. TNF is highly over expressed in both sera and renal tissue of the lupus mice and the levels of TNF is correlated with the degree of inflammatory organ disease. As the antagonist of TNF-α, PGRN was over-expression in SLE sufferer. So we think that PGRN is also a pathogenic factor of SLE. Besides, active SLE patients have higher PGRN serum levels compared with that treated with prednisone, which lead us to speculate that PGRN expression is correlated with disease activity.

Serine proteases can digest PGRN into individual granulin units, which are actually pro-inflammatory and can neutralize the anti-inflammatory effects of intact PGRN [11,12]. Both neutrophil elastase and PR3 digest PGRN to liberate granulin units [11,12]. In our study, we also found that the level of PR3 is higher in SLE patients both before and after prednisone treatment. Therefore the increases of PGRN detected in serum maybe result from the upregulation of individual PGRN units. PGRN may function as a pro-inflammatory factor. Further studies are needed to investigate the form of PGRN in the serum of SLE patients. In post- treatment SLE group, PGRN expression was lower than that in pre-treatment group, but the level of PR3 showed no change. Therefore, we suppose that PGRN could be used as a molecular marker of SLE.

In the pre-treatment group, both IL-6 and ds-DNA are higher than the post-treatment group and control group. They are both correlated of linear with the levels of PGRN (P < 0.05). IL-6 has been identified as an important factor in the pathogenesis of SLE [15]. The biological activities of IL-6 were diverse, inducing differentiation of B cells in plasma cells and production of IgG [16]. IL-6-deficient Mrl/lpr mice show a delayed onset of lupus nephritis and prolonged survival [17]. Murine lupus models indicate the involvement of IL-6 in B-cell hyperactivation and the onset of SLE [18,19]. The upregulation of IL-6 levels we observed was in accordance with the above reports. IL-6 is also associated with lupus nephritis and joint damage [20-22]. As IL-6 exerts both systemic and local effects, IL-6 targeting therapy has been proved to be effecient in inflammatory autoimmune diseases [23]. It has been shown that patients with active SLE have increased serum level of IL-6, which was correlated with disease activity or anti-dsDNA levels [20,21]. Therefore, we analyzed the relationship between IL-6 and PGRN levels and showed that they are correlated. Development of SLE is a very complex process. Therefore, we need more objective information to assess the disease activity in order to better guide clinical diagnosis. PGRN will be a very useful factor for the diagnosis of SLE. Therefore, PGRN was of value not only in the diagnosis, but also in the assessment of SLE severity.

GCs are powerful anti-inflammatory and immunosuppressive agents. They are widely used in systemic autoimmune diseases, such as SLE, dermatomyositis and other systemic diseases [24]. GCs exert their anti-inflammatory and immunosuppressive roles by two different mechanisms: the genomic pathway and the non-genomic pathway [4]. The interaction of GCs and GC receptor (GR) complex modulates gene expression to inhibit the transfer of leucocyte to inflammation site and damage the function of leucocyte, fibroblast and endothelial cells [3]. GCs reduce the synthesis of pro-inflammatory cytokines, such as IL-2, IL-6, TNF-α and prostaglandins (PGs) [24,25]. In SLE patients, disorder cytokine production induces immunodeficiency and leads to tissue inflammation and organ damage, such as diffuse proliferative lupus nephritis [26]. PR3 is involved in the development of a variety of autoimmune diseases, such as Wegener’s granulomatosis [27].

Our present study showed that the levels of PGRN, IL-6, TNFR, TNF-α and anti-dsDNA decreased after administration of large doses of prednisone 1 mg/kg/day for fourteen twenty-one consecutive days, and this was consistent with the above reports. In a word, the present study demonstrated that PGRN is up-regulated in both active and GC-treated SLE patients. PGRN was concerned to be correlated with the disease activity of SLE. Academic studies still needed to understand the precise mechanisms of PGRN in regulating SLE. Since TNFR signaling is involved SLE processes, antagonists of the TNF/TNFR pathway may lead to a new therapeutics for this disease. PGRN may be used as a diagnostic marker of systemic lupus erythematosus.

## Competing interests

The authors declare that they have no competing interests.

## Authors’ contributions

FQ did experiments, designed the experiments and wrote the manuscript; LS, FD, HL, QS, NY, and WL do experiments. XL designed the experiments and wrote the manuscript. All authors read and approved the final manuscript.
